# Dissecting cellular heterogeneity and intercellular communication in cholangiocarcinoma: implications for individualized therapeutic strategies

**DOI:** 10.3389/fgene.2023.1241834

**Published:** 2024-01-04

**Authors:** Zun-Qiang Zhou, Yi Zhang, Zi-Yang Xu, Xiao-Li Tang, Xiao-Hua Chen, Jiao Guan, Zheng-Yun Zhang

**Affiliations:** ^1^ Department of Surgery, Shanghai Sixth People’s Hospital Affiliated to Shanghai Jiao Tong University School of Medicine, Shanghai, China; ^2^ Department of Infectious Diseases, Shanghai Sixth People’s Hospital Affiliated to Shanghai Jiao Tong University School of Medicine, Shanghai, China

**Keywords:** cholangiocarcinoma, single-cell RNA sequencing, T cell, fibroblas, individualized therapy approach

## Abstract

**Background:** Cholangiocarcinoma is characterized by significant cellular heterogeneity and complex intercellular communication, which contribute to its progression and therapeutic resistance. Therefore, unraveling this complexity is essential for the development of effective treatments.

**Methods:** We employed single-cell RNA sequencing (scRNA-seq) to investigate cellular heterogeneity and intercellular communication in cholangiocarcinoma and adjacent normal tissues from two patients. Distinct cell types were identified, and gene ontology analyses were conducted to determine enriched pathways. Moreover, cell-cell communications were analyzed using CellChat, a computational framework. Additionally, we performed sub-clustering analysis of T cells and fibroblasts.

**Results:** The scRNA-seq analysis revealed distinct cell clusters and diverse cellular compositions of cholangiocarcinoma. CellChat analysis underscored an amplified outgoing signal from fibroblasts within the tumor, suggesting their pivotal role in the tumor microenvironment. Furthermore, T cell sub-clustering analysis revealed an active immune response within the tumor and new tumor-specific T cell clonotypes, suggesting scope for targeted immunotherapies. Moreover, fibroblast sub-clustering analysis indicated distinct functional states and highlighted the role of activated fibroblasts in shaping intercellular communication, particularly via CD99 and FN1 signaling.

**Conclusion:** Our findings reveal the intricate cellular heterogeneity and dynamic intercellular communication in cholangiocarcinoma, providing valuable insights into disease progression and potential therapeutic strategies.

## 1 Introduction

Intrahepatic cholangiocarcinoma (ICC) is a relatively rare yet notably aggressive form of primary liver tumors. It is the second most common liver malignancy and accounts for 5%–10% of all malignancies (Gupta and Dixon, 2017). Over the past decade, ICC has emerged as a significant global concern owing to a rise in its diagnostic incidence and mortality rates (Peery et al., 2019). ICC is characterized by its heterogeneity and distinct genetic and genomic features and can be found anywhere within the intrahepatic biliary tree, leading to different survival outcomes. Unfortunately, the majority of patients with ICC are diagnosed at an advanced stage, rendering surgical resection, which is an otherwise effective approach, infeasible (Rizvi and Gores, 2017). In such cases, palliative chemotherapy becomes the only applicable option. However, the recurrence rate remains high even after surgical resection, with a 5-year survival rate of 5%. Poor prognosis and the lack of available treatment options for unresectable ICC indicate the aggressive and chemotherapy-refractory nature of this disease (Kelley et al., 2020).

Cell-cell communication is regulated by biochemical signaling and governs individual cell processes and intercellular relationships. It is crucial for the overall tissue structure and function. ICC is characterized by remarkable cellular heterogeneity, and although the single-cell RNA sequencing (scRNA-seq) technology has enabled an unprecedented exploration of this diversity (Zhang et al., 2020; Ma et al., 2021; Alvisi et al., 2022; Bao et al., 2022; Song et al., 2022), ICC research has not yet fully utilized the potential of scRNA-seq to elucidate the complexities of cell-cell interactions and T cell receptor diversity in the disease context. Novel approaches that quantitatively analyze intercellular communication networks using scRNA-seq data have been developed to elucidate complex cell-cell interactions. Tools, such as CellChat, can be used to identify major cellular signaling inputs and outputs, classify signaling pathways, and extract intricate signaling patterns, thereby assisting in the detection of novel intercellular communications and the creation of cell-cell communication atlases across various tissues (Jin et al., 2021), offering unprecedented scale and depth (Almet et al., 2021). Furthermore, T cells play a crucial role in the tumor microenvironment. However, comprehensive profiling of their receptor repertoire at the single-cell level in ICC has not been performed.

In this study, we performed scRNA-seq to examine the intratumoral heterogeneity in two patients diagnosed with ICC. We conducted a thorough analysis of tumor and adjacent normal tissues to discern differences in cell-cell communication patterns between these two distinct regions. This analysis will provide a comprehensive landscape of cell-cell communication within ICC. In addition, to assess the role of T cells, we conducted an in-depth analysis of single-cell T cell receptor (scTCR) data. The comprehensive cell-cell communication and scTCR analyses will offer a new perspective of T cell receptor distribution and their dynamic interactions, further enriching our understanding of the complex cellular environment in ICC.

## 2 Methods

### 2.1 Sample collection and preparation

This study was conducted at the Department of Surgery, Shanghai Sixth People’s Hospital Affiliated to Shanghai Jiao Tong University School of Medicine. Two patients newly diagnosed with primary cholangiocarcinoma were enrolled between February and September 2022. The cohort comprised two males aged 66 and 81 years. All participants underwent common bile duct resection, partial liver resection, cholecystectomy, and choledochoenterostomy as the standard of care for cholangiocarcinoma. Pathological analysis revealed a moderately differentiated hilar cholangiocarcinoma with local metastasis, a moderately differentiated hilar cholangiocarcinoma with invasion into the mucosa and submucosa of the gallbladder, and a mixed neuroendocrine-non-neuroendocrine tumor consisting of a neuroendocrine tumor and tubular adenocarcinoma grade II-III with invasion into the gallbladder. Cholangiocarcinoma and adjacent normal tissues were collected from these two patients. The adjacent normal tissue samples were taken from the most distant site relative to the tumor border, usually >2 cm apart from the tumor edge. These tissues were carefully collected during the surgical procedure and immediately cleansed with sterile normal saline. Pathological analysis revealed two cases of moderately differentiated primary intrahepatic cholangiocarcinoma. The subsequent immunohistochemical examination confirmed the tumor’s characteristics. Each collected tissue was verified to be tumor and adjacent normal tissues by histopathological examination to ensure the integrity of the samples used in the study. Furthermore, to ensure preservation, they were stored in precooled MACS tissue storage solution at 4°C until analysis. This study was conducted according to the ethical guidelines set forth by the Ethical Committee of Shanghai Sixth People’s Hospital (2021-117-02), and all participants provided written informed consent.

### 2.2 scRNA-seq

Cholangiocarcinoma tissues were processed into single-cell suspensions for scRNA-seq. Briefly, the tissues were rinsed thrice in cold Dulbecco’s phosphate-buffered saline (Gibco) and delicately shifted to a pre-heated digestion buffer containing 2 mg/mL collagenase I, 2 mg/mL collagenase II, 0.9 U dispase, trypsin, and DMEM. The tissues were cut into small pieces in the digestion buffer and gently agitated in a 37-degree metal heater-shaker for 15 min. Then, a sample (10 µL) from the resuspension was analyzed using a hemocytometer.

In addition, the cell suspension was filtered using a 70 µm mesh filter, and subsequently washed with 5 mL DMEM. The cells were centrifuged (500 × *g*, 5 min, and 4°C), and the supernatant was discarded. The cells were then resuspended in 50–100 µL DMEM supplemented with 10% phosphate-buffered saline. The cell concentration was adjusted to 700–1200 cells/µL and then loaded onto a 10x Genomics Chromium machine. All samples were confirmed to have at least 80% viability using trypan blue staining.

Next, Gel Bead-In Emulsions were generated and used for reverse transcription and barcoding. The first-strand cDNA was purified with magnetic beads. After quality control check and quantification, cDNA was used for library construction using the 10x Chromium Single Cell 5′ Reagent kit and Chromium Single Cell Human TCR Amplification Kit (10x Genomics, Pleasanton, CA, United States), according to the manufacturer’s protocol. The prepared library was sequenced using the Illumina NovaSeq platform.

### 2.3 scRNA-seq analysis

The generated scRNA-seq data were processed using the Cell Ranger software (10x Genomics). Quality control, normalization, and identification of variable genes were performed using the Seurat R package (v4.3.0) (Butler et al., 2018). Cutoff for cells was set to nCount_RNA >2000, nCount_RNA <30000, and percent. mt < 10. Uniform manifold approximation and projection (UMAP) was used for data visualization.

Cells were clustered based on their gene expression profiles, and cell types were identified based on the expression of known marker genes. The relative proportions of cell types in tumor and adjacent normal tissues were also estimated. Additionally, copy number variations, which indicate malignancy, were calculated using the inferCNV R package (v1.12.0). All the detailed methods were included in source code for full recapitulation of the data.

### 2.4 Differential gene expression and pathway analyses

Differential gene expression between patient samples was evaluated using the Wilcoxon rank-sum test. Volcano plots were generated using the EnhancedVolcano package (v1.14.0) to visualize differentially expressed genes (DEGs). Then, Gene ontology (GO) analysis of the DEGs was conducted to identify enriched biological processes and pathways using the R package ClusterProfiler (v4.4.4) (Yu et al., 2012).

### 2.5 Cell-cell communication analysis

The CellChat R package (v1.6.1) (Jin et al., 2021), a computational framework, was used to analyze cell-cell communication based on ligand-receptor interactions. Changes in signaling pathways between tumor and adjacent normal tissues, especially those involving fibroblasts, were highlighted.

### 2.6 Cell sub-clustering and clonality analyses

T cell populations were sub-clustered to identify different T cell subtypes, and T cell receptor (TCR) sequencing data were analyzed using scRepertoire (v1.7.0) (Borcherding et al., 2020) to identify T cell clonality and tumor-specific clonotypes.

## 3 Results

### 3.1 Overview of single-cell transcriptomic analysis of patients with ICC

**FIGURE 1:**
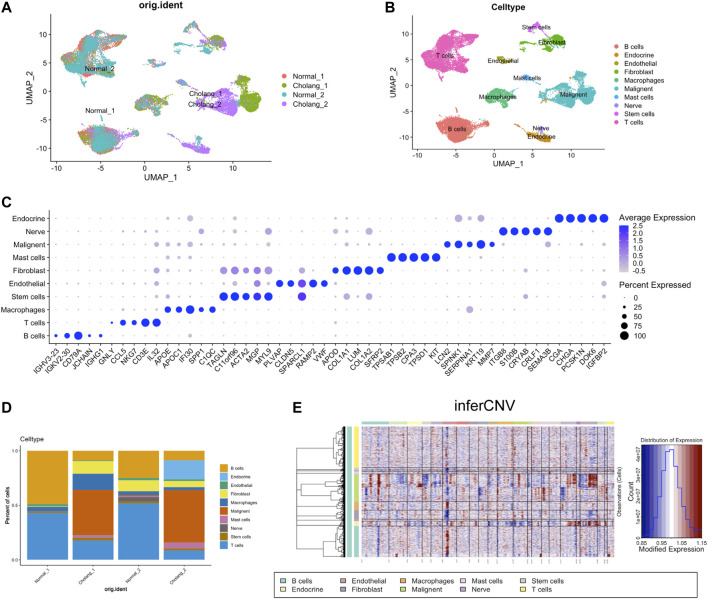
Single-cell analysis of patients with ICC. **(A)**. UMAP visualization of single-cell RNA sequencing data derived from tumor and adjacent normal tissues of two patients with cholangiocarcinoma. **(B)**. Cell type identification based on UMAP clusters and **(C)**. Corresponding marker genes. **(D)**. Proportional representation of cell types across different samples. **(E)**. Application of the InferCNV tool to determine the tumor cell population.

In this study, we investigated the cellular heterogeneity in cholangiocarcinoma and adjacent normal tissues from two patients using scRNA-seq. The resulting data were visualized using UMAP and found distinct cell clusters that represented different cell types within the tumor and surrounding normal tissues (Figures 1A, B). Notice that two tumors appear as relatively distinct cell clusters, suggesting the heterogeneity between two patients. The expression of established marker genes enabled the identification of several cell types, including malignant, immune, endothelial, endocrine, and stromal cells (Figure 1C). Moreover, by comparing the relative proportions of each cell type in tumor and adjacent normal tissue samples, we underscored the varied cellular composition and complexity in the cholangiocarcinoma landscape (Figure 1D). To validate the malignancy, we estimated the copy number variation score using inferCNV, which further strengthened our findings (Figure 1E). Within the inferCNV data, we observed high inferCNV scores within the endocrine cells, but they have shown distinct pattern compared to malignant cells. Based on our knowledge, classical markers shown in Figure 1C and inferCNV data, we have determined that malignant endocrine populations.

### 3.2 Distinct GO enrichment patterns observed in two patients

**FIGURE 2:**
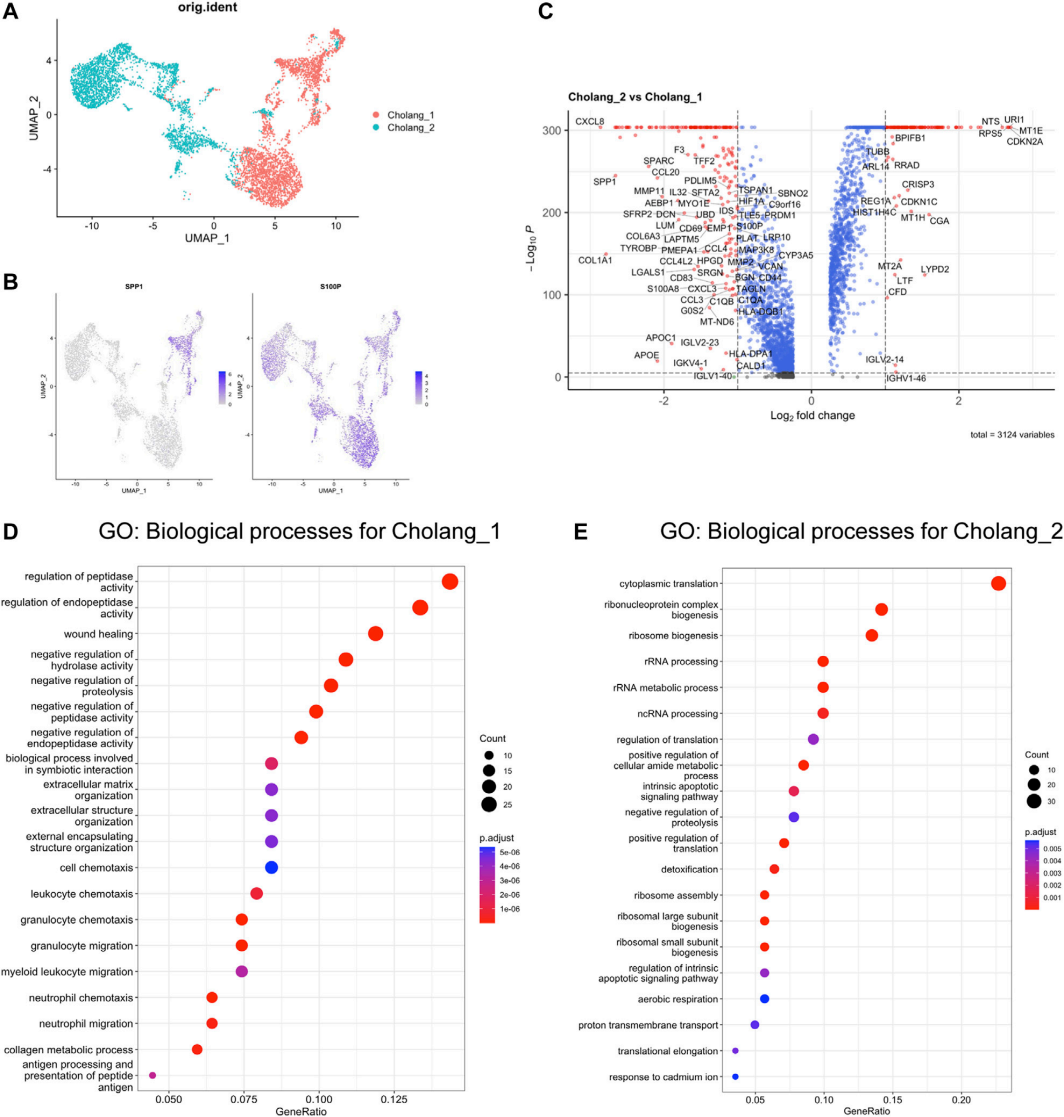
Distinct GO enrichment patterns were observed in two patients. **(A)**. UMAP-based clustering of malignant cells derived from tumors. **(B)**. Depiction of the distribution of key subtype markers, SPP1 and S100P, across the UMAP plot. **(C)**. Volcano plot illustrating differentially expressed genes, with those enriched in patient 2 (Log2FC > 1) displayed on the right and those enriched in patient 1 (Log2FC < 1) on the left. **(D, E)**. Top 20 pathways from the GO analysis of genes enriched in **(D)** patient 1 and **(E)** 2. Notably, patient 1 exhibited substantial enrichment of immune-related pathways, whereas patient 2 predominantly showed enrichment of cell cycle-related pathways.

To discern the differences between both the patients, we analyzed the heterogeneity among malignant cells in cholangiocarcinoma using scRNA-seq data. We leveraged UMAP clustering to unravel distinct subtypes within the tumor cells of each patient, thereby emphasizing the diversity of cancer cell populations (Figure 2A). Key subtype markers, SPP1 and S100P, were mapped across the UMAP plot to obtain valuable insights into their differential expression and potential roles in tumor progression (Figure 2B). As noted in previous studies (Song et al., 2022), these markers are attributed to various molecular subtypes. Consistently, our findings revealed exclusive expression of SPP1 in a sub-cluster of patient 1, while S100P was expressed across the malignant cells of both patients. To identify the DEGs between the two patients, we compared their gene expression profiles and visualized the data using a volcano plot (Figure 2C). The plot revealed unique gene expression signatures in the malignant cells of each patient, suggesting different molecular mechanisms underlying tumor progression. Notably, an abundance of cytokine/chemokine-related genes was identified in patient 1. Subsequently, we conducted GO analysis to highlight the top 20 enriched pathways in each patient (Figures 2D, E). This analysis showed significant enrichment of immune-related pathways in patient 1, suggesting a substantial role of immune regulation in the tumor. Consistently, the proportion of macrophages was increased in patient 1 (Figure 1D). Conversely, patient 2 exhibited an enrichment of cell cycle-related pathways, suggesting a possible variation in the tumor growth dynamics between the two patients. In summary, examination of the malignant cells in cholangiocarcinoma of two patients indicated distinct subtypes and unique gene expression patterns. The discrepancy in pathway enrichment, with patient 1 enriching immune-related pathways and patient 2 enriching cell cycle-related pathways, potentially demonstrates that several molecular mechanisms contribute to the progression of cholangiocarcinoma.

### 3.3 CellChat analysis underscores an amplified outgoing signal from fibroblasts in tumor tissues compared to adjacent normal tissues

**FIGURE 3:**
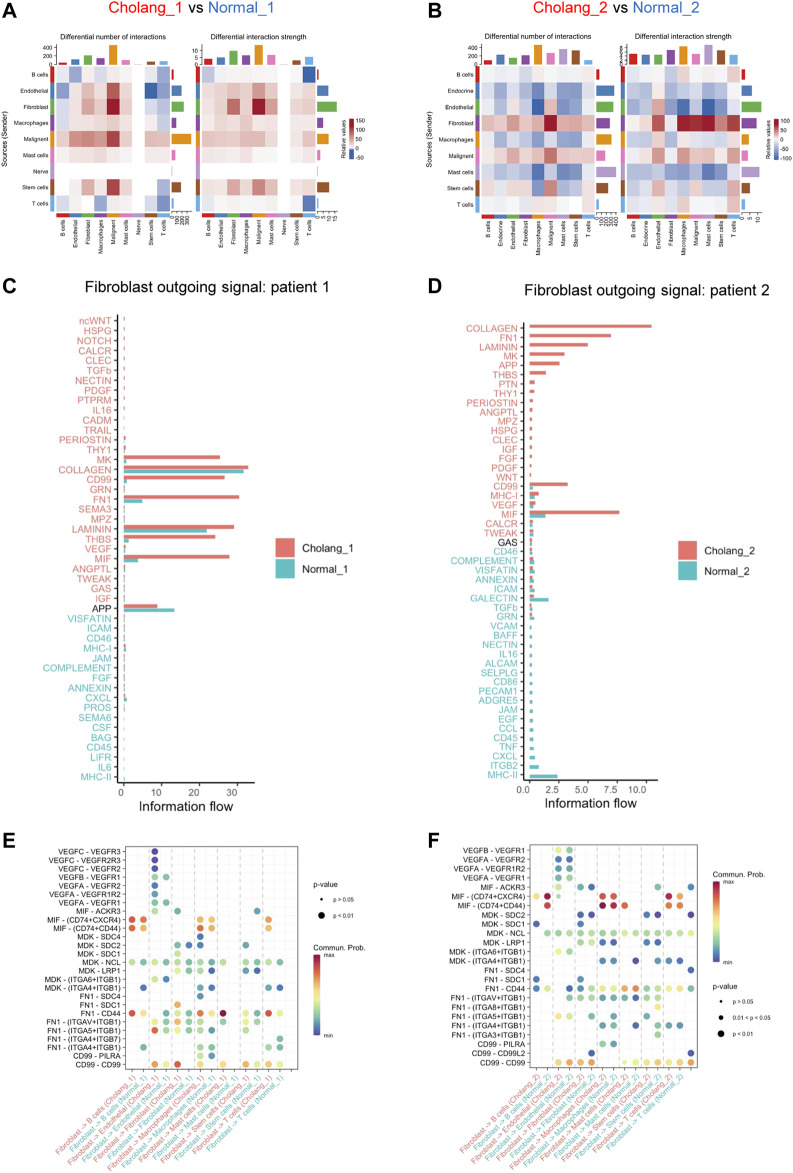
CellChat analysis underscores an amplified outgoing signal from fibroblasts in the tumor tissue compared to the adjacent normal tissue. **(A, B)**. CellChat analysis indicating alterations in cell-cell communication between tumor and adjacent normal tissues. An increase in tumor signaling is represented in red, while blue denotes an decrease in tumor tissue signaling. **(C, D)**. Detailed pathway enrichment in fibroblasts, indicating differences in tumor and normal tissues. **(E, F)**. An illustration of changes in specific ligand-receptor signals from fibroblasts and other cell types.

We subsequently employed CellChat to conduct a comprehensive analysis of changes in cell-cell communication between tumor and adjacent normal tissues. The alterations in cellular crosstalk between the two distinct environments are depicted in Figures 3A, B. This comparison showed significant alterations in cell-cell communication networks within the tumor microenvironment, with fibroblast outgoing signals being particularly amplified. Given the relatively stable fibroblast count in the tumor and normal tissues, our data suggest a heightened state of fibroblast activation in the tumor microenvironment. Moreover, pathway enrichment analysis, with a particular focus on fibroblasts, further highlighted the difference between the tumor and adjacent normal tissues (Figures 3C, D). This examination indicated marked differences in signaling pathways that are activated in tumor-associated fibroblasts compared to normal fibroblasts. Our findings suggest a pivotal role of tumor-associated fibroblasts in the modulation and activation of diverse signaling cascades, thereby possibly influencing the overall tumor microenvironment. Figures 3E, F illustrate specific ligand-receptor signal changes originating from fibroblasts and targeting other cells in the tumor microenvironment. This intricate landscape of cell-cell communication suggests the role of fibroblasts in regulating the interactive networks in the tumor and its periphery. Such interactions may significantly impact cancer cell behavior, potentially shaping therapeutic responses.

### 3.4 Sub-clustering T cells of all samples

**FIGURE 4:**
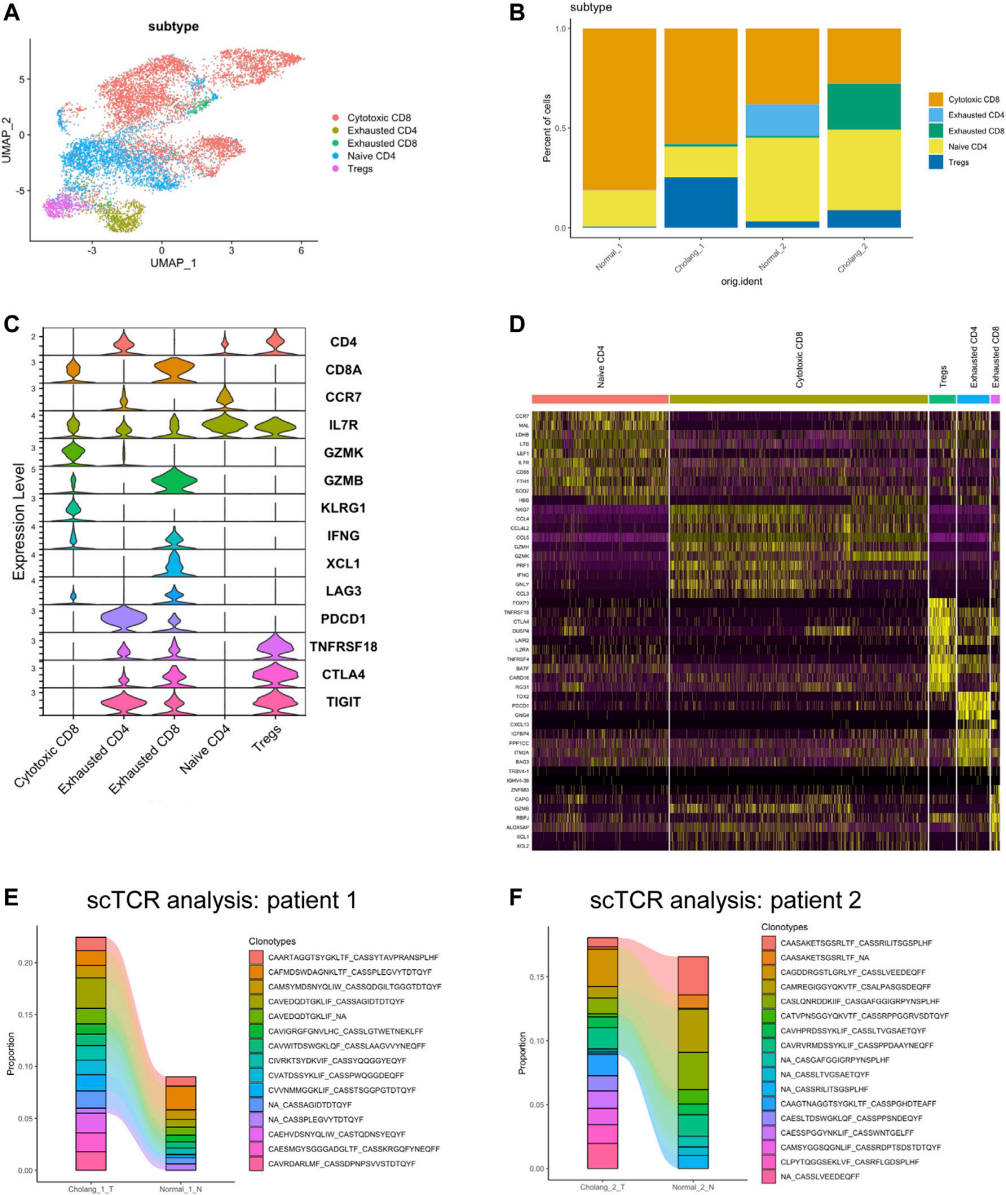
T cell analysis demonstrates a decrease in cytotoxic CD8 T cells in tumor tissues and a reduction in clonotypes. **(A)**. Sub-clustering of T cells across all samples. **(B)**. Quantitative representation of the proportion of different T cell subtypes. **(C)**. Expression of key markers in various T cell subtypes. **(D)**. Heatmap illustrating genes specific to different T cell populations. **(E, F)**. Single-cell T cell receptor analysis of the tumor and adjacent normal tissues, suggesting clonal expansion in the tumor and the emergence of new tumor-specific T cell clonotypes.

To confirm the intricate heterogeneity within T cell populations, we performed T cell sub-clustering analysis of all samples (Figure 4A). The proportional representation of different T cell subtypes revealed substantial variation across patients and sample locations, with an increase in exhausted and regulatory T cells and a decrease in cytotoxic T cells in the tumor. Figure 4C illustrates the expression of key markers in different T cell subtypes, while Figure 4D shows cluster-specific genes expressed in various T cell populations. To understand T cell clonality, we employed scTCR sequencing of tumor and adjacent normal tissues (Figures 4E,F). The results suggest clonal expansion within the tumor and the emergence of new, tumor-specific T cell clonotypes. These findings suggest an active immune response within the tumor microenvironment, emphasizing the potential role these specific T cell clonotypes might play in tumor immunosurveillance and progression. The observed clonal expansion and identification of tumor-specific T cell clonotypes indicate the dynamic interplay between the tumor and immune response, providing potential avenues for targeted immunotherapeutic strategies for cholangiocarcinoma.

### 3.5 Activated fibroblasts enriched in tumor samples contribute to elevated CD99 and FN1 signaling

**FIGURE 5:**
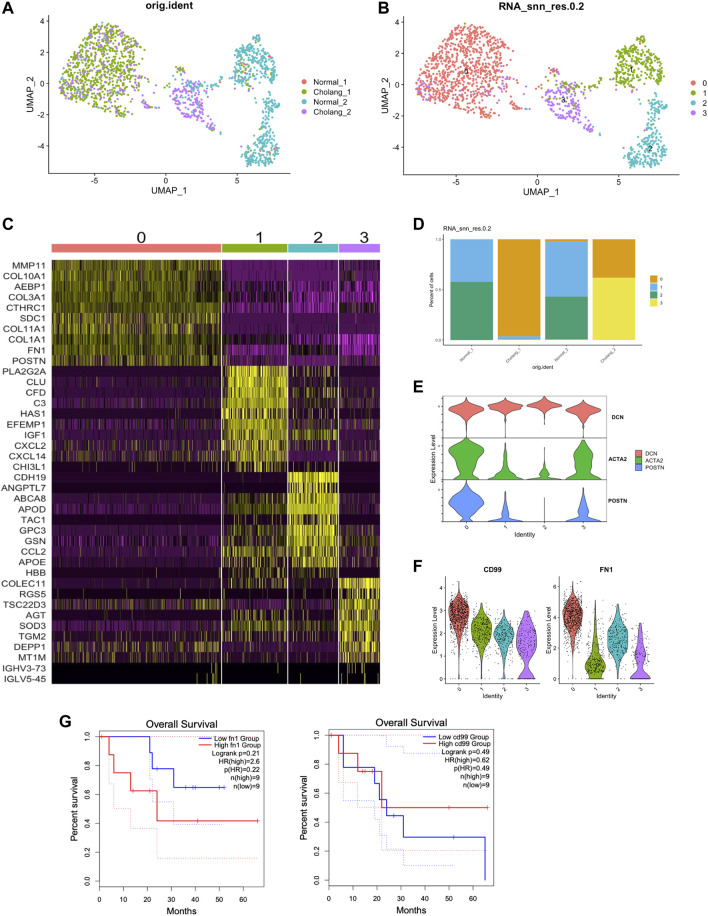
Activated fibroblasts enriched in tumor samples contribute to elevated CD99 and FN1 signaling. **(A, B)**. Sub-clustering of the fibroblast population from the samples resulted in the identification of four distinct clusters. **(C)**. Genes specific to different subtypes of fibroblasts, with POSTN marking activated fibroblasts and CLU marking resting fibroblasts. **(D)**. Relative proportions of the fibroblast subtypes. **(E)**. Enrichment of activated fibroblast markers in clusters 0 and 3. **(F)**. Elevated expression of CD99 and FN1 in cluster 0, aligning with the CellChat analysis above. **(G)**. Survival analysis of TCGA data based on FN1 and CD99 expression.

To further analyze the intricacies of fibroblasts, we performed sub-clustering analysis of fibroblast populations, and found four distinct clusters. This shows the variability among fibroblasts in the tumor microenvironment and neighboring normal tissues (Figures 5A, B). Figure 5C illustrates the genes uniquely expressed in the fibroblast subtypes, providing markers that can help distinguish between the functional states of fibroblasts in the samples. The relative proportions of these diverse fibroblast subtypes are shown in Figure 5D, shedding light on their distribution within the tumor and adjacent normal tissues. The classification of the fibroblasts into subtypes accentuates the profound impact of the tumor microenvironment on fibroblast activation. In particular, levels of activated fibroblast markers, a-SMA (ACTA2) and POSTN, in clusters 0 and 3 were considerably elevated (Figure 5E). Moreover, the expression of CD99 and FN1 in cluster 0 was increased, which is consistent with the CellChat results, reinforcing the supposition that these fibroblasts could be involved in modulating cell-cell communication in the tumor microenvironment (Figure 5F). This characterization of fibroblast clusters significantly enhances our understanding of the complex panorama of tumor microenvironments and lays a robust groundwork for future therapeutic strategies.

## 4 Discussion

In this study, we used scRNA and scTCR sequencing to determine diversity in ICC at a single-cell resolution, with a particular focus on cell-cell communication and TCR profiling. A previous study identified S100P and SPP1 as markers for different ICC subtypes and found distinct cellular environments and survival rates associated with each subtype (Song et al., 2022). They proposed that ICC can be classified into S100P + SPP1− and S100P−SPP1+ ICC subtypes based on S100P and SPP1 expression and noted significant differences in clinicopathological characteristics, gene regulatory networks, and immune infiltration between these two ICC subtypes. Our results revealed a new ICC subtype: S100P + SPP1+ (patient 1); the ICC of patient 2 aligned with the previously suggested S100P + SPP1− subtype. However, only a portion of patient 1’s tumor are SPP1+, which requires further classification and analysis with more patients. Furthermore, our GO analysis results of patient 1 indicated that the S100P + SPP1+ subtype may have distinct GO enrichment terms, such as immune response and chemotaxis-related pathways. Future studies should validate the distinct immune characteristics of the S100P + SPP1+ ICC subtype.

Understanding cell-cell communication is fundamental to deciphering the microenvironment of ICC. Previous studies have demonstrated that cancer-associated fibroblasts (CAFs) located in the tumor core and microvascular region are pivotal drivers of ICC progression. They cause this effect through high expression of microvasculature signature genes and IL-6, along with their intimate interaction with ICC cells via the IL-6/IL-6R pathway, which in turn modulate tumor epigenetic alterations (Zhang et al., 2020). In our study, a more holistic view of cell-cell communication showed an increase in overall outgoing signals from fibroblasts in tumor tissues compared to those of adjacent normal tissues. This indicates that CAFs may influence tumor cells and other cell types via the IL-6/IL-6R pathway and a wide range of outgoing signals.

The CD99 gene encodes a transmembrane protein that plays a crucial role in cell differentiation, adhesion, migration, and protein trafficking, with a distinct expression on the surface of hematopoietic cells in both the myeloid and lymphoid lineages (Ali et al., 2022). CD99 marks malignant myeloid stem cells (Kingwell, 2017), and recently, it was reported as a potent indicator of cancer stem cells, presenting a potentially effective therapeutic target in these malignancies (Pasello et al., 2018). Furthermore, fibronectin 1 (FN1) has been shown to facilitate the migration and invasion of various cancers, including papillary thyroid cancer, colon cancer, and clear cell renal cell carcinoma (Wang et al., 2022). However, the potential involvement of FN1 in modulating tumor immunity remains a relatively unexplored domain. Our results showed that both CD99 and FN1 (only found in one of the patients) are notably overexpressed in activated fibroblasts in ICC. Therefore, CD99 does not appear to function merely as a cancer stem cell marker, but also influences cholangiocarcinoma cell communication with the fibroblast and tumor microenvironment. Similarly, FN1 also showed substantial expression in activated fibroblasts. We performed survival analysis on TCGA cholangiocarcinoma (CHOL) patients based on FN1 and CD99 expression. Although without statistical significance, higher FN1 group has a trend of worse overall survival, suggesting potential diagnostic value of FN1 expression.

Evaluation of the prognosis for two patients with local metastasis suggested that CD99 and FN1 could serve as prognostic markers for ICC. However, the sample size is limited in our current study. Therefore, future research should aim to validate this hypothesis using larger cohorts and examine the correlation between CD99 and FN1+ fibroblasts and patient prognosis. Given that CD99 has been recognized as a therapeutic target in disease stem cells in myeloid malignancies (Chung et al., 2017), it may be beneficial to explore clinical trials that target CD99 in ICC. This could provide a promising avenue for improving ICC treatment strategies.

## Data Availability

The raw sequence data reported in this paper have been deposited in the Genome Sequence Archive (Genomics, Proteomics & Bioinformatics 2021) in National Genomics Data Center (Nucleic Acids Res 2022), China National Center for Bioinformation / Beijing Institute of Genomics, Chinese Academy of Sciences (GSA-Human: HRA006253) that are publicly accessible at https://ngdc.cncb.ac.cn/gsa-human/

## Abbreviations

ICC, intrahepatic cholangiocarcinoma; scRNA-seq, single-cell RNA sequencing; scTCR, single-cell T cell receptor; UMAP, Uniform manifold approximation and projection; DEGs, differentially expressed genes; GO, Gene ontology; TCR, T cell receptor.
